# Supplementary material for: HIVprotI: an integrated web based platform for prediction and design of HIV proteins inhibitors
Source: J Cheminform. 2018 Mar 9;10:12. doi: 10.1186/s13321-018-0266-y (PMC5845081; doi:10.1186/s13321-018-0266-y)
Supplement: Supplementary file 2 — Additional file 2. Source code of HIVProtI web server. [file 13321_2018_266_MOESM2_ESM.zip › HIVprotI_Source-code/hivprotI/pI_predict.php]

HIVprotI Pred


include("head.php");
?


#### Percentage Inhibition Prediction

Following is the  JSME editor , through which user can draw input of its desired chemical compound, or paste input in the form of SMILES, MOL and SDF format and subsequently predict its Percentage Inhibition of the selected HIV proteins.

  

  

  
  

**Draw your molecule:**

Your web browser must have JavaScript enabled
in order for this application to display correctly.

  
  

| Reverse transcriptase |  | Protease |  | Integrase |  | CHECK ALL |

  

---


***Note***: The prediction will take 2-5 minutes........

include("foot.php");
?
